# Supplementary material for: Therapeutic Silencing of BCL-2 Using NK Cell-Derived Exosomes as a Novel Therapeutic Approach in Breast Cancer
Source: Cancers (Basel). 2021 May 15;13(10):2397. doi: 10.3390/cancers13102397 (PMC8156181; doi:10.3390/cancers13102397)
Supplement: Supplementary file 1 [file cancers-13-02397-s001.zip › cancers-1179247-supplementaryfigures.pdf]

cancers

Supplementary Materials: Therapeutic silencing of BCL-2 using NK cell-derived exosomes as novel therapeutic approach in breast cancer

Table S1. Receptor expression of breast cancer cell lines

Cell line	Estrogen receptor (ER)	Progesterone receptor (PR)	Her2/neu receptor (HER2)
MCF-7	+	+	-
T-47D	+	+	-
SKBR3	-	-	+
MDA-MB-231	-	-	-

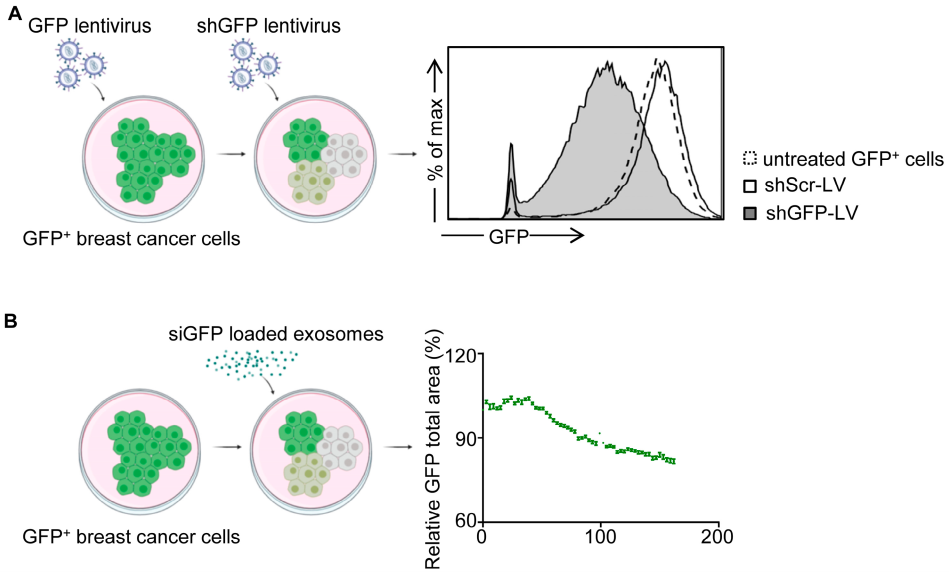

Figure S1. GFP silencing by siGFP NKExos. (**A**) Left, schematic workflow for LV transduction of GFP-expressing MDA-MB-231 cells. Supernatants of HEK293T cells producing LV carrying shGFP or shScr were used to transduce GFP⁺ MDA-MB-231 cells. Right, GFP⁺ MDA-MB-231 cells were treated with shGFP and shScr LVs or untreated, and GFP expression levels were determined by flow cytometry. (**B**) GFP⁺ MDA-MB-231 cells were treated with siGFP or siScr NKExos (200 µg/ml) over time and GFP expression was analyzed with an Incucyte live cell imaging system. Relative GFP signal was calculated by dividing total GFP area of siGFP NKExos treated cells by total GFP area of siScr NKExos treated cells.

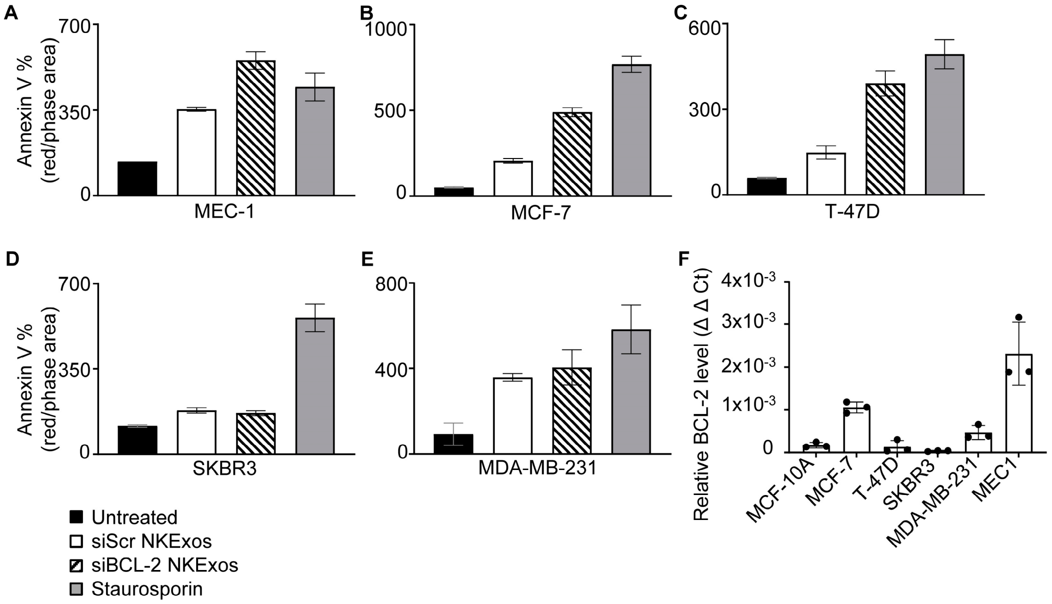

Figure S2. Apoptosis effects of siBCL-2 NKExos on CLL and breast cancer cells. (**A**) MEC-1, (**B**) MCF-7, (**C**) T-47D, (**D**) SKBR3, (**E**) MDA-MB-231 cells were treated with siBCL-2 or siScr NKExos (200 µg/ml) or staurosporine (2.5 µM), and Annexin V expression was determined by live cell imaging at 24 h. An exemplary result (n = 3, technical replicates) is shown for each cell lines. (**F**) Relative BCL-2 expression levels of MCF-10A, MCF-7, T-47D, SKBR3, MDA-MB-231 and MEC-1 cells are shown.
